# Optical detection of gadolinium(iii) ions *via* quantum dot aggregation†
†Electronic supplementary information (ESI) available: Fig. S1–S7, Tables S1–S4 and Movies (CdTe680_MCS_Movie.avi and CdTe680_Gd_MCS_Movie.avi). See DOI: 10.1039/c7ra03969g



**DOI:** 10.1039/c7ra03969g

**Published:** 2017-05-11

**Authors:** Steven D. Quinn, Steven W. Magennis

**Affiliations:** a WestCHEM, School of Chemistry, University of Glasgow, University Avenue, Glasgow, G12 8QQ, UK. Email: steven.magennis@glasgow.ac.uk

## Abstract

A rapid, sensitive and selective optical readout of the presence of gadolinium(iii) ions would have a wide range of applications for clinical and environmental monitoring. We demonstrate that water-soluble CdTe quantum dots (QDs) are induced to aggregate by Gd^3+^ ions in aqueous solution. By using a combination of photoluminescence spectroscopy, dynamic light scattering and fluorescence correlation spectroscopy (FCS) to monitor quantum dot aggregation kinetics, we correlate the efficiency of the self-quenching process with the degree of aggregation across a broad range of conditions, including different sizes of QDs. We attribute the aggregation to metal binding to the QD's surface ligands and the quenching to intra-aggregate energy transfer between QDs. When the strategy was applied to additional trivalent ions, the aggregation rate varied according to the particular trivalent metal ion used, suggesting that the selectivity can be enhanced and controlled by appropriate design of the capping ligands and solution conditions.

## Abstract 2

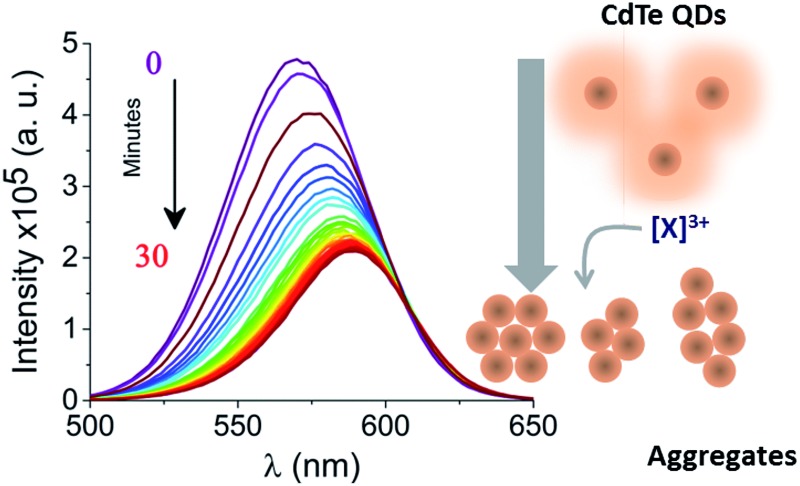
CdTe quantum dot aggregation induced by trivalent metal ions is followed using fluorescence, dynamic light scattering and fluorescence correlation spectroscopy.

## 

Gadolinium(iii) ions complexed to multidentate organic ligands have found widespread use as MRI contrast agents.^1^ However, there is now growing evidence that Gd^3+^ can accumulate *in vivo*, and lead to a range of adverse symptoms, including nephrogenic systemic fibrosis and inhibition of Ca^2+^-dependent enzymes (due to the similar size of Ca^2+^ and Gd^3+^).^2^ Although it is well established that Gd^3+^ is toxic and can interact with physiological systems, there is a distinct lack of knowledge about the cause and development of Gd^3+^-related diseases. Contrast agents have high thermodynamic and kinetic stability *in vitro*, but the speciation of the metal ion *in vivo*, including the role of processes such as transmetalation, dissociation, and ligand destruction is unclear.^3^

There is a need for new methods to test for Gd^3+^ in clinical samples, and also to examine their post-excretion distribution in the environment, particularly in waste and surface water. As recently reviewed,^4^ most methods for detecting Gd^3+^ have involved purification by HPLC or capillary electrophoresis combined with mass spectrometry or elemental analysis. However, there have also been reports of optical methods of analysis: for example, absorption changes upon ligand binding^5^ and gold nanoparticle aggregation,^6^ and a fluorescence-based DNA assay.^7^ New analytical methods that offer fast readout and that are cheap and easy to implement would be highly desirable for multiple applications, provided that they offer adequate selectivity and sensitivity.

We recently reported the interaction of trivalent metal ions, including Gd^3+^, with single isolated CdTe quantum dots (QDs) in an agarose gel.^8^ QDs have received widespread attention due to attractive properties^9,10^ such as their size-dependent tuneable emission, photostability and broad excitation spectra and we were interested in the effects of the metal ions on QD photophysics at the single-particle level. Recently, there have been reports of the quenching and aggregation of QDs induced by multivalent cations; Ca^2+^ was shown to cause aggregation, which was attributed to electrostatic screening of the CaCl_2_ as an electrolyte,^11^ while aggregation due to Mg^2+^, Ca^2+^, and Al^3+^ was attributed to binding of the metal to surface ligands and subsequent charge neutralisation.^12^ In fact, we had previously immobilized QDs in agarose to avoid potential aggregation of the QDs.^8^ We now report a study of CdTe QD aggregation at nanomolar concentrations in aqueous solution in the presence of trivalent ions (Fig. 1a) and demonstrate that this approach shows promise as a selective sensor for heavy trivalent metal ions, including Gd^3+^.

**Fig. 1:**
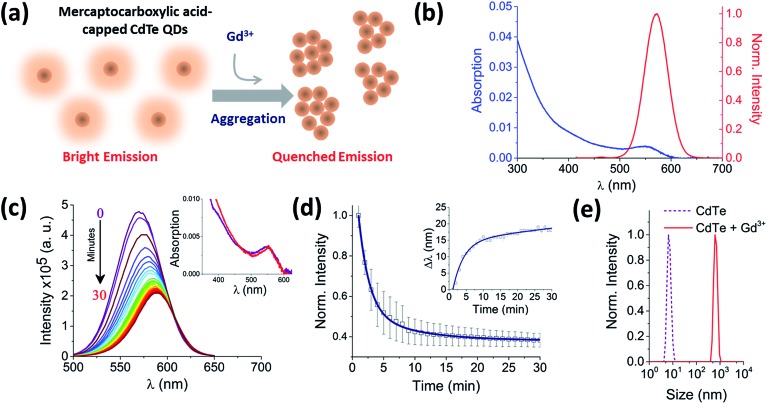
Real-time probing of CdTe 580 QD aggregation induced by Gd^3+^. (a) The injection of a trivalent metal nitrate solution (10 μM Gd^3+^) alters the ensemble fluorescence emission properties of 25 nM CdTe 580 (pH 8) as aggregation occurs. (b) Absorption (blue) and fluorescence emission spectrum with *λ*_exc_ = 400 nm (red) of 25 nM CdTe 580 QDs at pH 8. (c) Representative fluorescence quenching of 25 nM CdTe 580 QDs induced to aggregate over 30 minutes by addition of 10 μM Gd^3+^. Inset: variation in absorption spectra between the start (*t* = 0 min, purple) and end (*t* = 30 min, red) of the aggregation process. (d) The corresponding variation in fluorescence intensity and red shift (inset) across the 30 minute time window are shown. (e) DLS size distributions of CdTe 580 QDs prior to the injection of 10 μM Gd^3+^ and at the endpoint (*t* = 30 min) of the aggregation process.

## Experimental

### Materials

Trizma-hydrochloride (Tris–HCl), aluminium nitrate nonahydrate, gadolinium nitrate hexahydrate, yttrium nitrate hexahydrate, lutetium nitrate hydrate, sodium chloride (Fisher Scientific, UK), potassium nitrate, and rhodamine 110 were used without further purification; chemicals from Sigma-Aldrich (UK) unless stated otherwise. Core-type CdTe quantum dots with emission centered on 530 nm, 580 nm and 680 nm, termed CdTe 530, CdTe 580 and CdTe 680, respectively, were purchased from Plasmachem (Germany) and used without further purification. Buffer solutions were produced using Milli-Q ultrapure water (Millipore, UK).

### Addition of metal salts to QD solutions

A stock solution of *ca.* 3 mM of metal salt in buffer was prepared and a small amount (*ca.* 10 μL) added into 3 mL of the QD solution to minimize dilution effects; spectra were corrected for this dilution. The typical final concentration of metal salt was in the micromolar range as indicated in the main text.

### Optical spectroscopy

Absorption spectra of quantum dots in aqueous solution (20 mM Tris–HCl buffer, pH 8) were measured with a Cary 50 (Agilent Technologies) spectrophotometer. Final sample concentrations of CdTe 530, CdTe 580 and CdTe 680 were determined using extinction coefficients of 60 000 M^–1^ cm^–1^ at 496 nm, 150 000 M^–1^ cm^–1^ at 550 nm and 211 000 M^–1^ cm^–1^ at 644 nm, respectively. Corrected emission spectra from quantum dot solutions were collected under magic angle conditions using a Fluoromax fluorescence spectrophotometer (Horiba Scientific).

Data analysis was carried out using laboratory-written routines developed in Origin 8.0. Emission spectra were recorded every minute for time-dependent measurements. Intensity–time trajectories were constructed by integrating the fluorescence intensity over the full emission spectrum.

### Dynamic light scattering

Quantum dot size distributions were measured at the end-point of the metal-ion induced aggregation process at 21 °C using dynamic light scattering (Zetasizer, Malvern Instruments, UK) with 633 nm (4 mW) incident light. The fluctuating scattering intensities were detected using an avalanche photodiode detector at 90° to the incident light and autocorrelated to generate a correlation function. The aggregate size is reported as the hydrodynamic diameter, extracted from the Stokes–Einstein equation.

### Fluorescence correlation spectroscopy (FCS)

FCS and multichannel scalar (MCS) measurements were performed on a home-built confocal microscope with excitation at 488 nm as described previously.^13^ The average laser power of the focused beam at the sample was *ca.* 170 μW. All measurements are reported for a temperature of 22 ± 1 °C.

Correlation curves, *G*(*τ*),were fitted according to eqn (1) or (2). Both equations account for diffusion in a 3D Gaussian volume; eqn (1) also includes a term for triplet deactivation, while eqn (2) has a stretched exponential term which has been used previously to model CdTe quantum dots.^14^
1
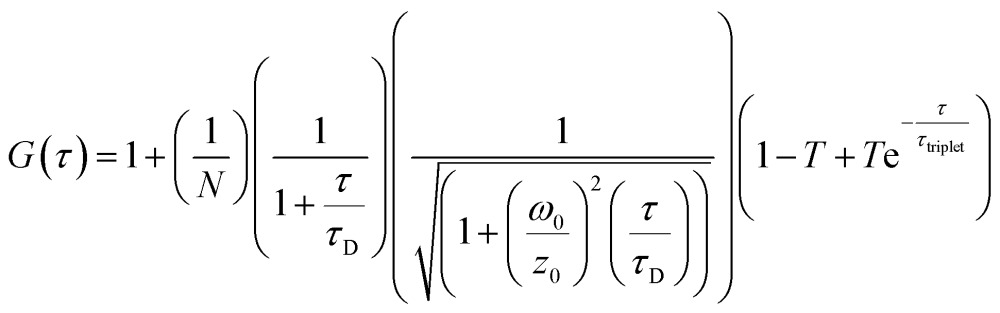
where *τ* is the lag time, *N* is the number of molecules in the confocal volume, *τ*_D_ is the translational diffusion time, *ω*_0_ and *z*_0_ are the distances at which the 3D Gaussian volume has decayed to 1/e^2^ in the *x*/*y* and *z* directions, respectively, *T* is the triplet fraction and *τ*_triplet_ is the triplet lifetime.2
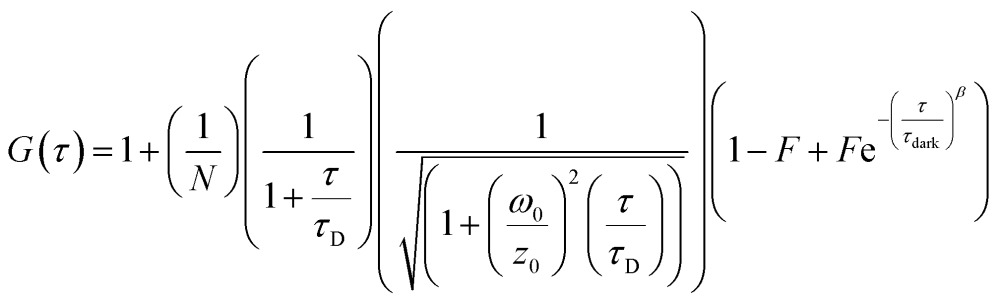
where *F* is the fraction of the dark state, *τ*_dark_ is the dark state relaxation time, and *β* is a stretching factor.

The translational diffusion time is related to the diffusion coefficient, *D*, *via* eqn (3).3
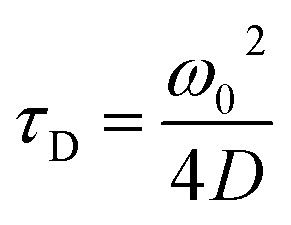



FCS curves for rhodamine 110 (diffusion coefficient in water is 4.4 × 10^–10^ m^2^ s^–1^ at 22.5 °C (ref. 15)) and the CdTe QDs under identical conditions were fitted to eqn (1) and (2), respectively. The diffusion coefficient of the QDs could then be calculated (eqn (3)). The hydrodynamic radius of the diffusing particle, *R*_H_, can then be found *via* the Stokes–Einstein relation (eqn (4)).4
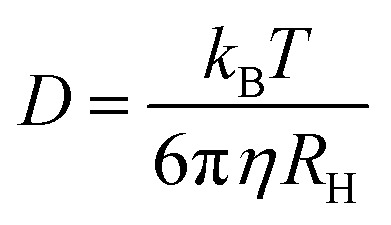
where *k*_B_ is Boltzmann's constant, *T* is the temperature of the medium and *η* is the dynamic viscosity of the medium.

## Results and discussion

Hydrophilic CdTe QDs were characterized using ensemble absorption and fluorescence spectroscopy in 20 mM Tris–HCl buffer at pH 8. The absorption and emission spectra were typical for QDs (Fig. 1b). We showed previously that the same CdTe 580 QDs were stable in solution at pH 8 and insensitive to minor pH changes.^8^ At pH 8, the carboxylate groups of the ligands (TGA and TGA-related mercaptocarboxylic acid ligands) on the QD surface make the quantum dots negatively charged. The addition of trivalent metal ions as nitrate salts to a solution of CdTe QDs induces the rapid and reproducible formation of QD aggregates (Fig. 1). For example, 10 μM Gd^3+^ added to a solution containing 25 nM CdTe 580 induced a quenching of the fluorescence spectra (60% decrease) (Fig. 1c) concurrent with a 17 nm red-shift over a 30 minute time window (Fig. 1c and d inset and S1†). In contrast, the absorption spectrum showed little change following addition of Gd^3+^ (Fig. 1c inset). The fluorescence quenching trajectory was fitted to a bi-exponential decay with average rate of 0.83 ± 0.05 s^–1^ (Fig. 1d, Table 1). Under the same conditions, dynamic light scattering was performed before and after addition of 10 μM Gd^3+^. Prior to injection of Gd^3+^, the QDs displayed a size distribution centered on 6 nm, but after incubation with 10 μM Gd^3+^ for 30 minutes, a homogeneous diameter centered on 650 nm was obtained (Fig. 1e). Importantly, the injection of Gd^3+^ was performed at 0.4% (v/v). As demonstrated in our earlier work^8^ and by others,^16^ minor dilutions of ligand-capped QDs (*e.g.* 1 : 10) can lead to the formation of small aggregates (∼50 nm) *via* a mechanism that is thought to involve rearrangement and rapid washout of the surface ligands.

**Table 1:** Pre-exponential factors and rate constants associated with the fluorescence quenching trajectories of 25 nM CdTe 580 in the presence of 10 μM Gd^3+^, 10 μM Y^3+^, 10 μM Lu^3+^ and 10 μM Al^3+^ (pH 8). Kinetic parameters were obtained from individual non-linear least squares fits of the fluorescence trajectories to exponential functions of the form *I*(*t*) = *y*_0_ + *A*_1_e^–*t*/*t*_1_^ + *A*_2_e^–*t*/*t*_2_^, where *t*_1_ and *t*_2_ are time constants with amplitudes *A*_1_ and *A*_2_ observed over time, *t*

	Al^3+^	Lu^3+^	Y^3+^	Gd^3+^
*y*_0_	0.87 ± 0.01	0.60 ± 0.02	0.28 ± 0.01	0.38 ± 0.01
*A*_1_	0.11 ± 0.01	0.14 ± 0.02	0.57 ± 0.01	0.86 ± 0.02
*t*_1_ (s)	3.36 ± 0.17	2.19 ± 0.36	1.45 ± 0.03	1.15 ± 0.07
*A*_2_	—	0.26 ± 0.01	0.15 ± 0.01	0.17 ± 0.03
*t*_2_ (s)	—	4.69 ± 3.67	5.67 ± 0.5	5.55 ± 0.08
*k*_1_ (s^–1^)	0.29 ± 0.01	0.45 ± 0.08	0.69 ± 0.01	0.86 ± 0.05
*k*_2_ (s^–1^)	—	0.21 ± 0.01	0.18 ± 0.01	0.18 ± 0.02
***k***_**av**_ **(s** ^**–1**^ **)**	***0.29 ± 0.01***	***0.23* ± 0.01**	***0.66 ± 0.01***	***0.83 ± 0.05***
*χ* ^2^ ^*a*^	0.995	0.997	0.999	0.999

^*a*^Numbers represent the values obtained for the goodness of the fit expressed as reduced chi-square (*χ*_r_^2^) calculated following the equation 
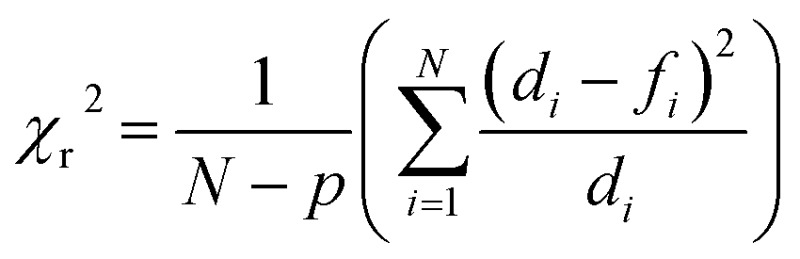
 where N represents the number of data points, *p* the number of fitting parameters, *d*_*i*_ the experimental data and *f*_*i*_ the fitting result.

**Fig. 2:**
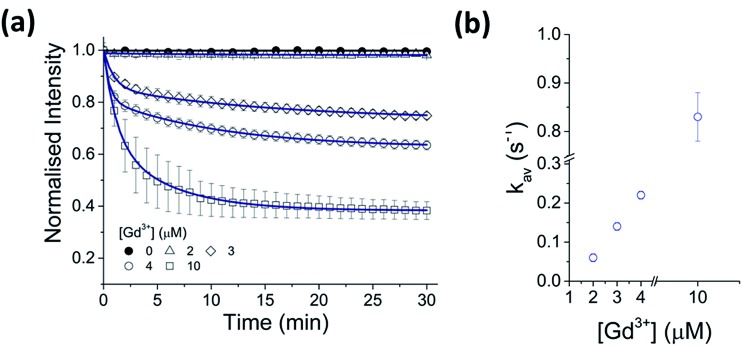
Kinetics of CdTe 580 quenching induced by Gd^3+^. (a) Normalized variation in the fluorescence intensity of CdTe 580 QDs as a function of time in the absence and presence of 2 μM, 3 μM, 4 μM and 10 μM Gd^3+^. The solid lines represent fits to linear (black) and exponential decay (blue) functions. (b) The corresponding variation in the average quenching decay rate as a function of Gd^3+^ concentration. Data are expressed as the mean ± SEM.

A strong dependence of Gd^3+^ concentration on the rate of CdTe 580 quenching was observed when identical samples were incubated with 2, 3 and 4 μM Gd^3+^, respectively (Fig. 2, Table S1†). To ensure reproducibility of the quenching rates, different batches of CdTe 580 were tested and negligible batch-to-batch variations were observed (Fig. S2, Table S2†).

**Fig. 3:**
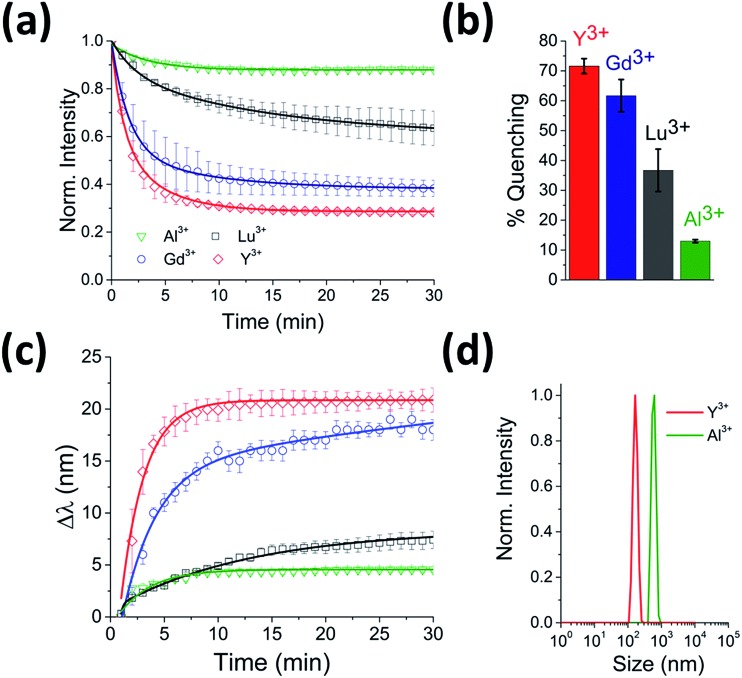
Real-time probing of CdTe 580 QD aggregation induced by Y^3+^, Lu^3+^ and Al^3+^. (a) Representative fluorescence quenching trajectories of 25 nM CdTe 580 QDs induced to aggregate over 30 minutes by addition of 10 μM Y^3+^ (red), 10 μM Lu^3+^ (black) and 10 μM Al^3+^ (green) at pH 8. For comparison, the 10 μM Gd^3+^ (blue) from Fig. 1d is also shown. (b) Comparative bar plot summarizing the relative variations in fluorescence quenching observed at *t* = 30 minutes. (c) The corresponding red-shifts in emission spectra (*λ*_exc_ = 400 nm) across the 30 minute time window are shown. (d) DLS size distributions of CdTe 580 QDs after injection (*t* = 30 min) of 10 μM Y^3+^ (red) and 10 μM Al^3+^ (green).

To test for any dependence of the aggregation phenomenon on the metal ion, we performed equivalent experiments with smaller (Al^3+^ and Y^3+^) and larger (Lu^3+^) trivalent ions and with monovalent ions (K^+^ and Na^+^). When CdTe 580 was incubated with monovalent ions (KNO_3_ and NaCl) at the same ionic strength, no effect on the quantum dot emission (Fig. S3 and S4†) was observed and no aggregation was detected *via* DLS (Fig. S5†) under the conditions tested. In contrast, when 10 μM Y^3+^ was added to 25 nM CdTe 580, the fluorescence was quenched in a similar manner to the Gd^3+^ experiment, with bi-exponential quenching (Fig. 3a, Table 1) and a similar quenching magnitude of 70% after 30 minutes (Fig. 3b). However, when 10 μM Al^3+^ was added to an identical sample of CdTe 580 the quenching trajectories displayed mono-exponential exponential behaviour, and a 6-fold reduction in quenching magnitude was observed with 3-fold reduction in the average rate (Fig. 3a and b, Table 1). When 10 μM Lu^3+^ was added to an identical CdTe 580 sample the quenching was again biexponential but with average rate of quenching that was closer to that of the much smaller Al^3+^ ion, rather than the similarly-sized Gd^3+^ (Fig. 3a and b, Table 1). In all cases, quenching occurred simultaneously with red-shifts in the emission spectra over similar timescales (Fig. 3c). Dynamic light scattering confirmed the presence of aggregates. These were 200 nm diameter structures in the case of Y^3+^, and 600 nm diameter aggregates with Al^3+^ (Fig. 3d).

Importantly, although the quenching efficiency obtained from repeated experiments consistently displayed ion-induced quenching of the emission spectra (Y^3+^ > Gd^3+^ > Lu^3+^ > Al^3+^) (Fig. 3b), changes in the absorption spectra after addition of the same ions were negligible (Fig. 1c inset). Aggregation-induced quenching is normally attributed to either ground-state electronic coupling (which should alter the absorption spectrum as well as the emission spectrum) or energy transfer within aggregates from QDs with larger bandgap to those with smaller bandgap.^17^ In the case of energy transfer, one would expect quenching and a concomitant red-shift, as observed, but without a requirement for changes to the absorption spectrum. Therefore, based on the available information, we propose that an energy-transfer quenching mechanism is operational.

**Fig. 4:**
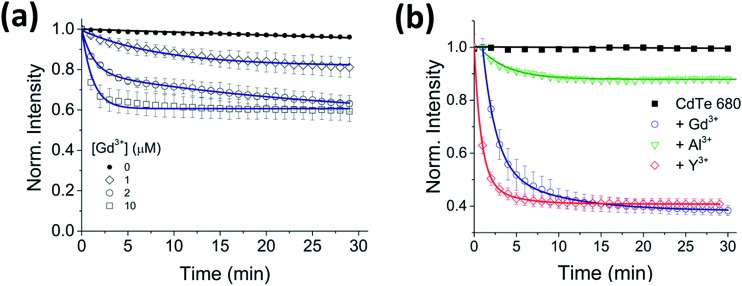
Effect of trivalent metal ions on CdTe 530 and CdTe 680 emission. (a) Normalized variation in the fluorescence intensity of 25 nM CdTe 530 QDs as a function of time in the absence and presence of 1 μM, 2 μM and 10 μM Gd^3+^. The solid lines represent fits to linear (black) and mono-exponential decay (blue) functions. (b) Representative fluorescence quenching trajectories of 25 nM CdTe 680 QDs induced to aggregate over 30 minutes by addition of 10 μM Gd^3+^ (blue), 10 μM Y^3+^ (red) and 10 μM Al^3+^ (green) at pH 8. All data are expressed as the mean ± SEM.

To investigate the generality of the ion-induced aggregation and quenching, the fluorescence response of QDs with different sizes, and therefore emission wavelengths, was explored. When Gd^3+^ was incubated with CdTe 530 at pH 8, similar quenching behavior (Fig. 4a) was observed, with the rate of aggregation varying 8-fold across the conditions tested (Table S3†). We note that the quenching trajectories were again concurrent with time-dependent red-shifts in the emission spectra (Fig. S6†). These characteristic optical signatures were also observed when 10 μM Gd^3+^ was added to a solution containing 25 nM CdTe 680 (pH 8) (Fig. 4b and S7, Table S4†). The addition of 10 μM Al^3+^ and 10 μM Y^3+^ to CdTe 680 also followed similar behavior to CdTe 580 (Fig. 4b and S7, Table S4†).

**Fig. 5:**
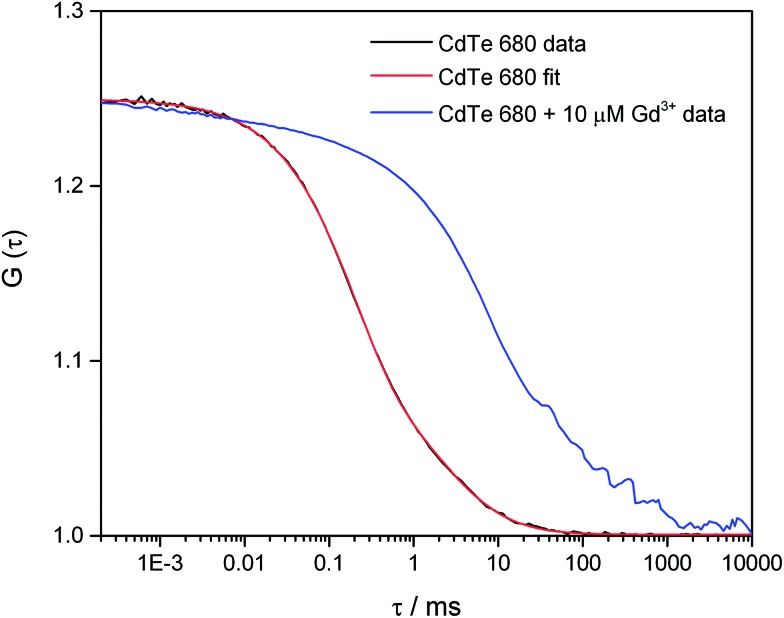
Fluorescence correlation spectroscopy of CdTe 680 QDs in the absence and presence of Gd^3+^. The correlation curve, *G*(*τ*) of 25 nM CdTe 680 QDs in the absence of Gd^3+^ (black line) can be fitted (red line) using eqn (2). The fitting parameters are *N* = 4.01, *τ*_D_ = 1.985 ms, *F* = 0.626, *τ*_dark_ = 0.197 ms, and *β* = 0.793. Addition of 10 μM Gd^3+^ results in a pronounced shift to longer lag times.

In order to investigate the aggregation by a complementary method to DLS, we used fluorescence correlation spectroscopy (FCS) to study CdTe 680 QDs in solution. FCS is an excellent method for studying the diffusion characteristics of nanoparticles with single-particle sensitivity.^14^ We first measured correlation curves for CdTe 680 QDs in buffer in the absence of metal ions (Fig. 5). It is well known that quantum dot blinking follows a power law distribution^8^ and that a model incorporating diffusion together with a stretched exponential term (eqn (2)) is able reproduce the correlation curves of CdTe,^14^ an approach that also works for other luminescent nanoparticles.^18^ We found that our data fitted well to the stretched exponential model (eqn (2)), with the best fits yielding a diffusion time of 1.80 ± 0.19 ms. Note that the curves could not be fitted to a simple model of diffusion alone, or diffusion with an additional exponential decay term (eqn (1)). By measuring rhodamine 110, which has a known diffusion coefficient in water, under identical experimental conditions (and fitted to eqn (1)), we use eqn (3) and (4) to calculate the hydrodynamic radius of the CdTe 680 QDs as 4.59 ± 0.44 nm. This is significantly larger than the manufacturers reported radius of 2.1 nm, which used UV-Vis spectroscopy referenced to TEM.^19^ FCS has been shown to result in larger radii than TEM for QDs (by *ca.* 20%), which can account for some of the difference.^20^ Since we were able to get good fits (by visual inspection) when the diffusion times were fixed at values around 1 ms, we believe that the discrepancy in size may reflect the presence of a small amount of low-order aggregates present in the absence of Gd^3+^.

In contrast, we found that following the incubation of CdTe 680 with 10 μM Gd^3+^, there was a pronounced shift of the correlation curve to longer times (Fig. 5). We attribute this to the presence of particles with much longer diffusion times, in comparison to the free QDs. There is likely to be a mixture of free QDs and aggregates (of varying sizes), leading to a range of diffusion times, together with additional fluctuations at short time (including QD blinking and intra-aggregate energy transfer). However, a simple model incorporating two diffusing species, one of which was fixed to the diffusion time recovered for the free QDs, gave a reasonable fit; the other diffusion time of *ca.* 70 ms corresponds to a hydrodynamic radius of *ca.* 160 microns.

As additional direct evidence of the QD aggregation, we also recorded multichannel scalar (MCS) traces of the fluorescence intensity *vs.* time as particles diffuse through the focused laser beam of the confocal microscope. As shown in the ESI,† Movies for CdTe 680 before addition of Gd^3+^ (CdTe680_MCS_Movie.avi) show background signal with the occasional intense burst of fluorescence of short duration (few milliseconds) due to diffusion of individual QDs. In contrast, addition of Gd^3+^ (CdTe680_Gd_MCS_Movie.avi†) results in bursts of much longer duration, characteristic of larger diffusing species, which we assign as QD aggregates.

Although all the trivalent ions investigated induced QD aggregation, we observed quite different aggregation rates for trivalent ions of similar size (Gd^3+^ and Lu^3+^), while also observing similar rates for ions of rather different sizes (Y^3+^/Gd^3+^ and Al^3+^/Lu^3+^). Similarly, the Al^3+^ and Gd^3+^ formed comparably-sized aggregates, yet had quite different aggregation kinetics. In contrast, although the kinetics for Gd^3+^ and Y^3+^ were similar, there was a three-fold difference in the average diameter of aggregates (by DLS). A previous study of Al^3+^-induced aggregation of CdTe QDs postulated a mechanism involving metal ion binding to surface ligands, resulting in surface charge neutralization or bridging between QDs.^12^ The ion-dependent data we have reported also suggests that the standard DLVO theory of colloidal interactions is not enough to explain our observations, and that surface layers play an important role.^21^ However, determination of the exact aggregation mechanism would require not only knowledge of the exact speciation of the aquated metal ions at a particular pH, but also of the binding constants of these metal species with the surface-bound ligands and the QD surface itself. The morphology and chemical structure of the aggregates may well also vary as a function of cation, which might be expected to result in different amounts of quenching. The lack of a clear trend in the aggregation data may even point toward the operation of more than one aggregation mechanism. It is possible that the metals themselves are not even incorporated in the aggregates but serve to remove the ligands from the surface of the QD, thereby promoting aggregation.

## Conclusion

In spite of the apparent complexity, this is a clear proof-of-principle demonstration that QD aggregation can be used as a sensor for the presence of heavy trivalent ions, including Gd^3+^, and that both the rates of aggregation (*via* fluorescence) and the size of aggregates (*via* DLS and FCS) may be used as measurands to distinguish a particular metal. We have demonstrated very promising selectivity and sensitivity with relatively cheap, commercially-available quantum dots, one particular group of metal salts (nitrates) and a small range of solution conditions. Therefore, we believe that there is considerable scope for the development of tailored nanoparticles, with optimized surface ligands and solution conditions for real-time monitoring of important metal ions in a wide range of applications.
