# Supplementary material for: Optical detection of gadolinium(iii) ions via quantum dot aggregation
Source: RSC Adv. 2017 May 11;7(40):24730–5. doi: 10.1039/c7ra03969g (PMC5735353; doi:10.1039/c7ra03969g)
Supplement: Supplementary file 3 [file RA-007-C7RA03969G-s003.pdf]

Electronic Supplementary Material (ESI) for RSC Advances.
This journal is © The Royal Society of Chemistry 2017

Supporting information for

Optical detection of gadolinium (III) ions via quantum dot aggregation

Steven D. Quinn[1,#] and Steven W. Magennis[1,*]

*Correspondence to Dr. Steven Magennis

This PDF includes:

Figures S1-S7

Tables S1-S4

Supporting text for the MCS Movies

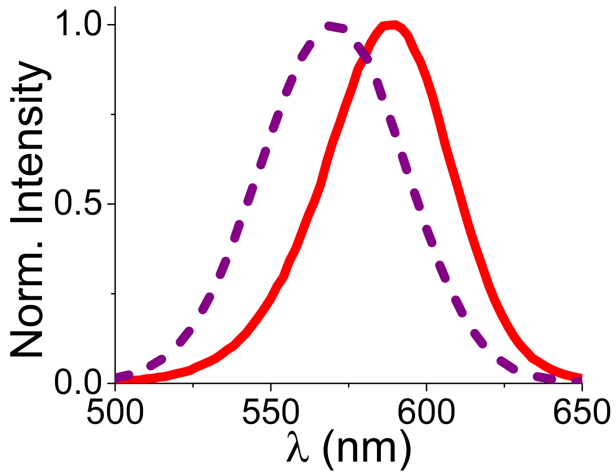

Figure S1. Normalized variation in CdTe 580 fluorescence emission spectra between the start (t=0 min, dashed) and end (t = 30 min, solid) of the Gd^{3+} induced aggregation process.

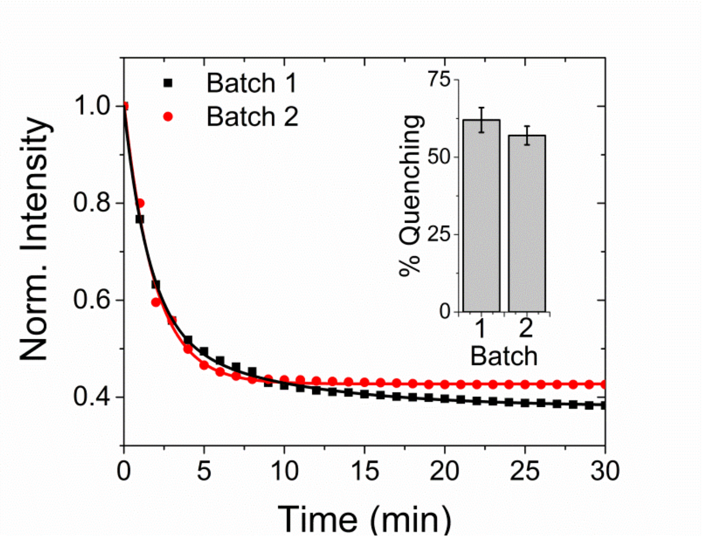

Figure S2. CdTe 580 quenching trajectories induced by Gd^{3+} display negligible batch-to-batch variation. Normalised variation in the fluorescence intensity of 25 nM CdTe 580 from two separate batches (red and black) as a function of time in the presence 10 µM $Gd(NO_3)_3.6H_2O$ in 20 mM Tris-HCl, pH 8 buffer. Inset: bar chart summarizing the quenching magnitude exhibited by two separated CdTe 580 batches after addition of 10 µM Gd^{3+} at t = 30 minutes.

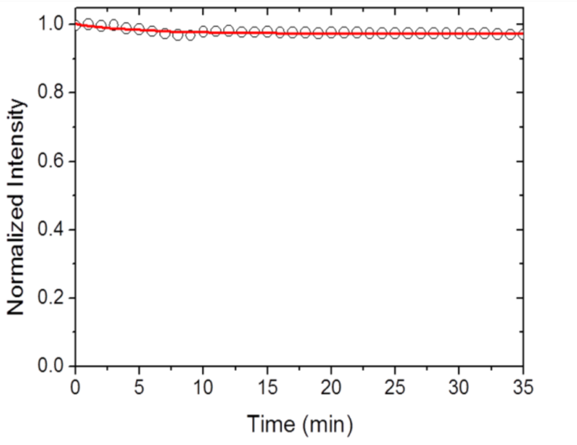

Figure S3. Effect of KNO_3 on CdTe 580 emission. (a) Normalized variation in fluorescence emission intensity of 25 nM CdTe 580 after injection of 120 μM KNO_3 at pH 8. λ_{exc}= 400 nm.

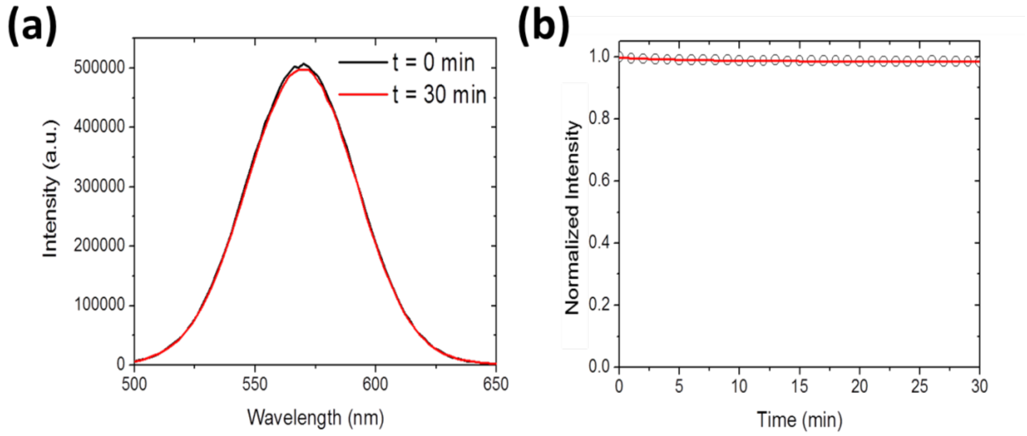

Figure S4. Effect of NaCl on CdTe 580 emission. (a) Normalized variation in fluorescence emission spectra of 25 nM CdTe 580 before (t = 0 min) and after (t = 30 min) injection of 120 μM NaCl at pH 8 with λ_{exc}= 400 nm. (b) The corresponding normalized variation in fluorescence intensity across the entire 30 minute time window.

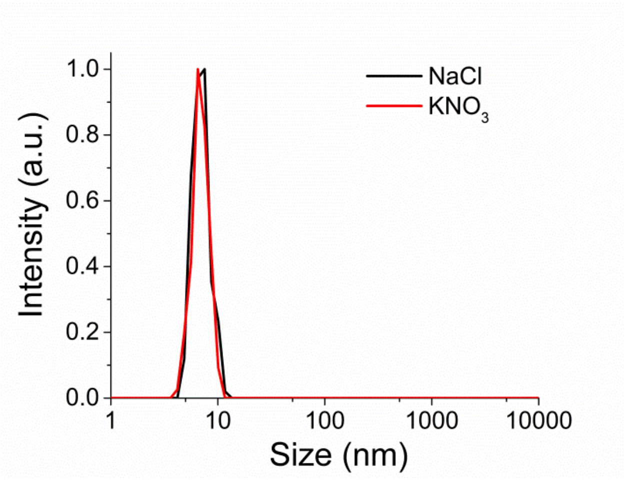

Figure S5. DLS size distributions of CdTe 580 QDs at t=30 minutes, after injection of 120 μM NaCl (black) and 120 μM KNO_3 (red) at pH 8.

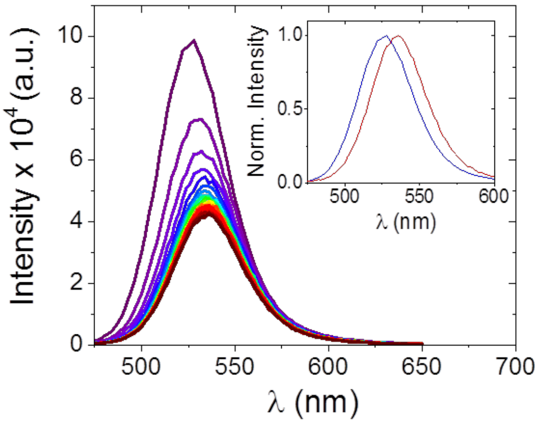

Figure S6. Fluorescence quenching of 25 nM CdTe 530 induced by addition of 10 μM Gd^{3+}. Fluorescence emission spectra of CdTe 530 in the presence of 10 μM Gd^{3+} in 20 mM Tris-HCl buffer (pH 8) with λ_{exc} = 400 nm followed over a 30 minute time window (purple to red). Inset: normalized variation in emission spectra between the start (t = 0 minutes, blue) and end (t = 30 minutes, red) of the quenching trajectory.

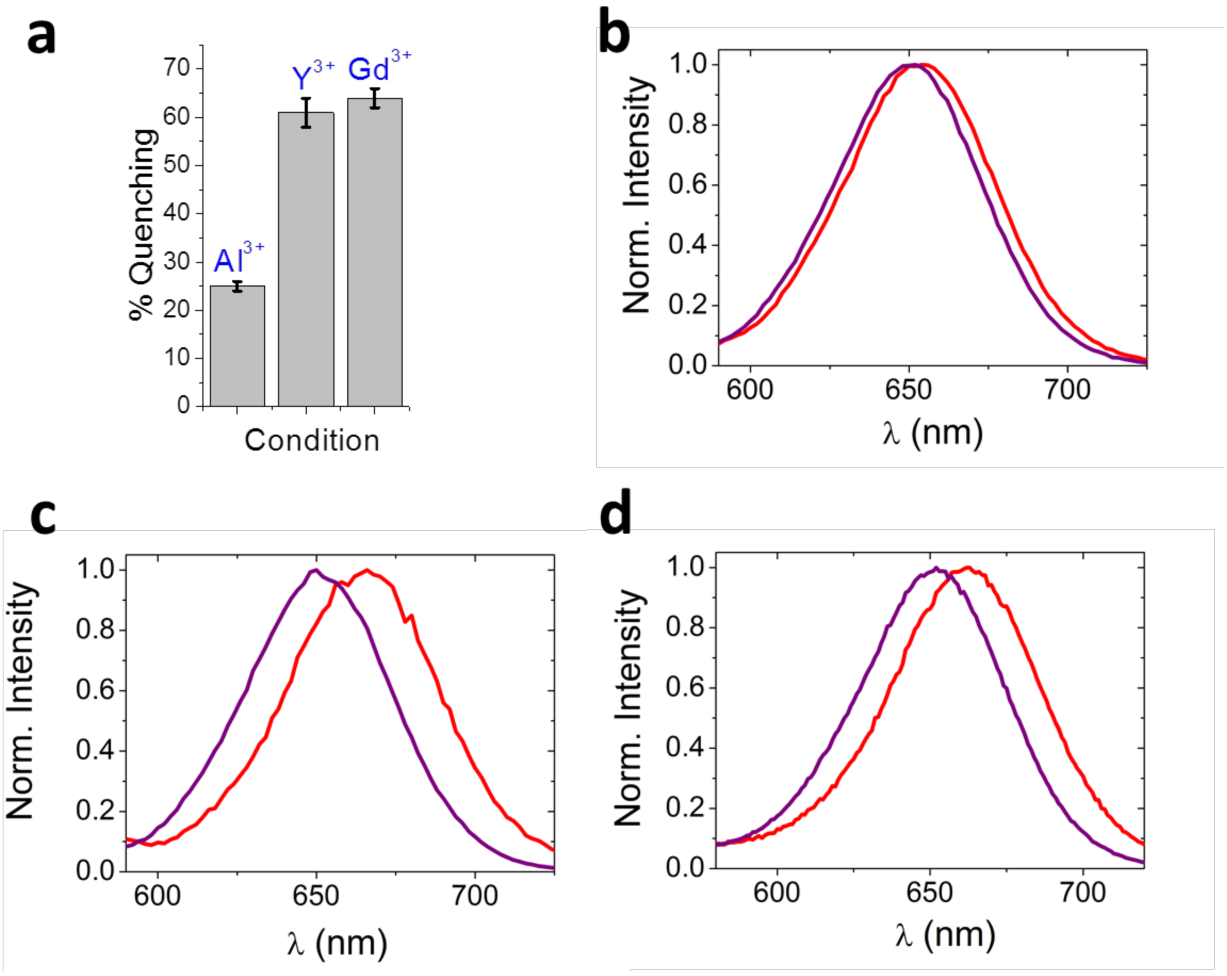

Figure S7. Quenching of CdTe 680 induced by 10µM Al^{3+}, 10µM Y^{3+} and 10µM Gd^{3+}. (a) Bar chart summarizing the quenching magnitude exhibited by 25 nM CdTe 680 after addition of 10 µM Al^{3+}, 10 µM Y^{3+} and 10 µM Gd^{3+} after 30 minutes incubation in 20 mM Tris-HCl buffer at pH 8. The corresponding normalized variation in emission spectra between the start (t = 0) and end (t = 30 minutes) of the quenching trajectories in the presence of (b) 10 µM Al^{3+}, (c) 10 µM Y^{3+} and (d) 10 µM Gd^{3+} are also shown.

Table S1 Pre-exponential factors and rate constants associated with the fluorescence quenching of 25 nM CdTe 580 in the presence of Gd^{3+} (pH 8). Kinetic parameters were obtained from individual non-linear least squares fits of the fluorescence trajectories to exponential functions of the form $I(t) = y_o + A_1e^{-t/t1} + A_2e^{-t/t2}$, where t_1 and t_2 are time constants with amplitudes A_1 and A_2 observed over time, t.

	**2 μM**	**3 μM**	**4 μM**	**10 μM**
y_0	0.98 ± 0.03	0.73 ± 0.02	0.63 ± 0.01	0.38 ± 0.01
A_1	0.05 ± 0.01	0.14 ± 0.01	0.26 ± 0.01	0.86 ± 0.02
t_1 (s)	15.11 ± 1.45	0.93 ± 0.06	0.61 ± 0.02	1.15 ± 0.07
A_2	--	0.12 ± 0.01	0.16 ± 0.02	0.17 ± 0.03
t_2 (s)	--	16.11 ± 1.26	10.48 ± 1.33	5.55 ± 0.08
k_1 (s^{-1})	0.06 ± 0.01	1.07 ± 0.06	1.63 ± 0.05	0.86 ± 0.05
k_2 (s^{-1})	--	0.08 ± 0.01	0.09 ± 0.01	0.18 ± 0.02
**k_{av} (s^{-1})**	***0.06 ± 0.01***	***0.14 ± 0.01***	***0.22 ± 0.01***	***0.83 ± 0.05***
**χ^2 ***	0.974	0.999	0.979	0.999

*Numbers represent the values obtained for the goodness of the fit expressed as reduced Chi-square (χ^2) calculated following the $\chi^2 = \frac{1}{N-p}\left(\sum_{i=1}^{N}\frac{(d_i - f_i)^2}{d_i}\right)$ where N represents the number of data points, p the number of fitting parameters, d_i the experimental data and f_i the fitting result.

Table S2. Pre-exponential factors and rate constants associated with the fluorescence quenching trajectories of 25 nM CdTe 580 from two separated batches in the presence of 10 μM Gd^{3+} (pH 8). Kinetic parameters were obtained from individual non-linear least squares fits of the fluorescence trajectories to exponential functions of the form $I(t) = y_o + A_1e^{-t/t1} + A_2e^{-t/t2}$, where t_1 and t_2 are time constants with amplitudes A_1 and A_2 observed over time, t.

	**Batch 1**	**Batch 2**
y_0	0.38 ± 0.01	0.42 ± 0.01
A_1	0.75 ± 0.02	0.79 ± 0.02
t_1 (s)	1.17 ± 0.01	1.19 ± 0.02
A_2	0.16 ± 0.02	0.18 ± 0.01
t_2 (s)	5.44 ± 0.07	5.49 ± 0.07
k_1 (s^{-1})	0.85 ± 0.01	0.84 ± 0.01
k_2 (s^{-1})	0.18 ± 0.01	0.18 ± 0.01
**k_{av} (s^{-1})**	***0.82 ± 0.01***	***0.81 ± 0.01***
χ^2 *	0.999	0.998

*Numbers represent the values obtained for the goodness of the fit expressed as reduced Chi-square (χ^2) calculated following the equation $\chi^2 = \frac{1}{N-p}\left(\sum_{i=1}^{N}\frac{(d_i - f_i)^2}{d_i}\right)$ where N represents the number of data points, p the number of fitting parameters, d_i the experimental data and f_i the fitting result.

Table S3. Pre-exponential factors and rate constants associated with the fluorescence quenching trajectories of 25 nM CdTe 530 in the presence of Gd^{3+} (pH 8). Kinetic parameters were obtained from individual non-linear least squares fits of the fluorescence trajectories to exponential functions of the form $I(t) = y_o + A_1e^{-t/t1}$ where t_1 is the time constant with amplitudes A_1 observed over time, t.

	**1 μM**	**2 μM**	**10 μM**
y_0	0.81 ± 0.01	0.55 ± 0.06	0.60 ± 0.02
A_1	0.18 ± 0.01	0.21 ± 0.01	0.39 ± 0.01
t_1 (s)	8.72 ± 0.07	2.76 ± 0.12	1.20 ± 0.03
k_1 (s^{-1})	0.11 ± 0.01	0.36 ± 0.02	0.83 ± 0.02
**χ^2 ***	0.988	0.996	0.998

*Numbers represent the values obtained for the goodness of the fit expressed as reduced Chi-square (χ^2) calculated following the equation $\chi^2_{\backslash} = \frac{1}{N-p}\left(\sum_{i=1}^{N}\frac{(d_i - f_i)^2}{d_i}\right)$ where N represents the number of data points, p the number of fitting parameters, d_i the experimental data and f_i the fitting result.

Table S4. Pre-exponential factors and rate constants associated with the fluorescence quenching trajectories of 25 nM CdTe 680 in the presence of 10 μM Gd^{3+} , 10 μM Al^{3+} and 10 μM Y^{3+} (pH 8). Kinetic parameters were obtained from individual non-linear least squares fits of the fluorescence trajectories to exponential functions of the form $I(t) = y_o + A_1e^{-t/t1} + A_2e^{-t/t2}$, where t_1 and t_2 are time constants with amplitudes A_1 and A_2 observed over time, t.

	**Gd^{3+}**	**Al^{3+}**	**Y^{3+}**
y_0	0.38 ± 0.01	0.88 ± 0.01	0.41 ± 0.01
A_1	0.86 ± 0.02	0.15 ± 0.01	0.49 ± 0.01
t_1 (s)	1.56 ± 0.07	3.36 ± 0.17	0.82 ± 0.02
A_2	0.19 ± 0.02	--	0.09 ± 0.01
t_2 (s)	7.83 ± 0.75	--	3.45 ± 0.26
k_1 (s^{-1})	0.64 ± 0.03	0.29 ± 0.02	1.21 ± 0.03
k_2 (s^{-1})	0.13 ± 0.01	--	0.29 ± 0.02
**k_{av} (s^{-1})**	***0.62 ± 0.03***	***0.29 ± 0.02***	***1.16 ± 0.03***
**χ^2 ***	0.999	0.951	0.999

*Numbers represent the values obtained for the goodness of the fit expressed as reduced Chi-square (χ^2) calculated following the equation $\chi^2 = \frac{1}{N-p}\left(\sum_{i=1}^{N}\frac{(d_i - f_i)^2}{d_i}\right)$ where N represents the number of data points, p the number of fitting parameters, d_i the experimental data and f_i the fitting result.

Multichannel Scalar (MCS) Movies

The MCS movies (CdTe680_MCS_Movie.avi and CdTe680_Gd_MCS_Movie.avi) were recorded with a confocal microscope with detection of the QD emission by 4 avalanche photodiode (APD) detectors. The green and red traces correspond to perpendicular and parallel polarisation of the QD emission after passing through a 710/130 nm bandpass filter; the blue traces for the other two APDs (for emission that has passed through a 525/50 nm bandpass filter) show only background and dark counts.
